# Development and validation of UPLC-MS/MS method for studying the pharmacokinetic interaction of dasabuvir and tamoxifen, 4-hydroxytamoxifen in Wistar rats

**DOI:** 10.1038/s41598-020-60613-2

**Published:** 2020-02-26

**Authors:** Aliyah Almomen, Hadir M. Maher, Nourah Z. Alzoman, Shereen M. Shehata, Shorog M. Al-taweel, Ashwaq A. Alanazi

**Affiliations:** 1 College of Pharmacy, Department of Pharmaceutical Chemistry, King Saud University, Riyadh, 11495 P.O. Box 22452, Saudi Arabia; 2 Faculty of Pharmacy, Department of Pharmaceutical Analytical Chemistry, University of Alexandria, El-Messalah, Alexandria, 21521 Egypt

**Keywords:** Chemistry, Bioanalytical chemistry

## Abstract

Hepatitis C virus (HCV) is the main cause of chronic hepatitis and probably liver cirrhosis. Dasabuvir (DSV) is a direct-acting antiviral agent with efficiency in managing HCV. The anti-viral activity of the anti-estrogen drug tamoxifen (TAM) suggested the synergistic effect of DSV and TAM for blocking the replication of HCV. However, being substrates and inhibitors of efflux transporters (TAM inhibits P-gp, DSV inhibits P-gp and BCRP), there is a possibility for a pharmacokinetic (PK) drug-drug interaction. In this work, a new UPLC-MS/MS method was developed and validated for the simultaneous determination of TAM, its active metabolite 4-hydroxy tamoxifen (TOH), and DSV in rat plasma. The method was applied to investigate the PK interaction between DSV and TAM/TOH following the co-administration of DSV and TAM to Wistar rats. Chromatographic analysis was performed on Waters BEH^TM^ C18 column using a mobile phase of acetonitrile/water containing 0.1% formic acid (80: 20, v/v). The method allowed the determination of concentration ranges 20–1000, 0.1–500, 0.5–500 ng/mL for DSV, TAM, and TOH, respectively. Unexpectedly, results revealed the absence of PK interactions between DSV and TAM/TOH, compared with their single administration, suggesting the safety of co-administering DSV/TAM as an anti-viral combination without the need of dosage adjustment.

## Introduction

**Figure 1:**
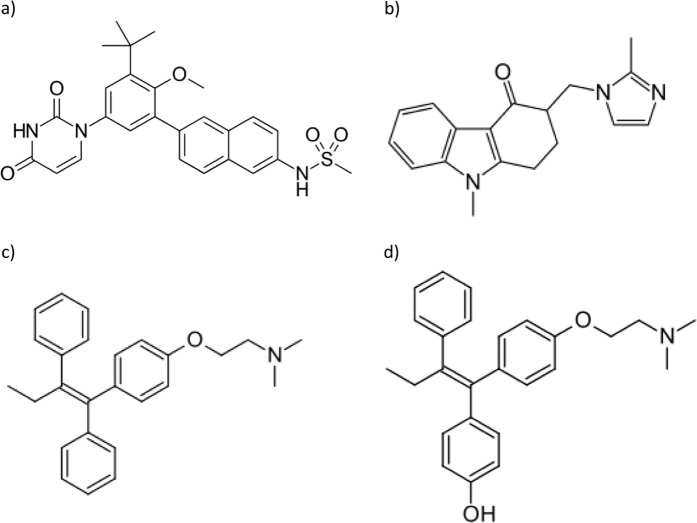
Chemical structure of the studied compounds, DSV, (**a**), OND, (**b**), TAM, (**c**), and TOH, (**d**).

Hepatitis C virus (HCV) is considered as the main cause of chronic hepatitis which mostly progress to liver cirrhosis, and probably hepatocellular carcinoma. HCV has also been associated with various extra-hepatic disorders, including kidney, thyroid, skeletal system, nervous system, and immunological disorders^1,2^. Treatment strategies of HCV infections aim to completely suppress the virus with further control of any hepatic and extra-hepatic health disorders. The emergence of direct-acting antiviral agents (DAAs) has greatly influenced the treatment of chronic HCV infections. These DAAs target different stages in the virus life cycle. Compared to the previous interferon-based regimens, DAAs have proven to be more effective, well-tolerated, and associated with less drug-related toxicities^3^. Dasabuvir (DSV), Fig. 1, is a non-nucleoside inhibitor that blocks the replication cycle of HCV by inhibiting the virus RNA encoded by the non-structural (NS) 5B gene. DSV has been approved by the US and EU agencies, whether alone or in combination with other antiviral agents, for the treatment of GT1a and GT1b HCV, even some cases of liver cirrhosis. The pharmacokinetic (PK) behavior of DSV has been thoroughly studied in both healthy subjects and those with HCV^4^. DSV is mainly metabolized by cytochrome P450 (CYP) 2C8 enzymes and to a lesser extent by CYP3A4, with no marked modulatory effect on the CYP enzymes^3–5^. DSV acts as both a substrate and an inhibitor of the efflux transport proteins, p-glycoprotein (P-gp) and breast cancer resistance protein (BCRP), in addition to uridine diphosphate glucuronosyltransferase (UGT) 1A1^3–5^.

Mechanistic data showed that DAAs are substrates/modulators of liver enzymes and/or active transporters which contribute largely to drug-drug interactions (DDIs). In this concern, DAAs can be either a “victim” where its PK parameters could be affected by co-administered drugs, or as the “perpetrator” by affecting the PK of other co-administered medications^3^. PK studies have been used to evaluate DDIs between DAAs with many non-HCV medications^3,4,6–9^. DSV exposure was reported to increase with CYP2C8 inhibitors and strong CYP3A and P-gp inhibitors^6–9^. On the other hand, strong CYP3A4 inducers resulted in a decrease in DSV exposure^4^. Also, an increase in rosuvastatin bioavailability was reported when co-administered with DSV. This was referred to BCRP inhibition induced by DSV^4^. However, no significant effect of DSV was recorded on the PK parameters of tacrolimus, cyclosporine^8^, and omeprazole^7^.

Tamoxifen (TAM), Fig. 1, is known as a selective estrogen receptor (ER) modulator used mainly in the management of ER-α positive breast cancer. The chemopreventive effect of TAM in many other cancers (e.g. hepatocellular carcinoma^10^, ……..) has been reported.

The association between estrogen receptors (ER) and HCV replication was described previously^11,12^. TAM inhibits viral RNA polymerase NS5B with a subsequent anti-HCV activity^11^. It is also noteworthy to mention that TAM has completed clinical trials (phase 1) for the treatment of HCV Infection^12^.

TAM has been considered as a prodrug which is activated inside the body by the CYP450 enzymes; CYP2D6, CYP2C9, and CYP3A4; to active metabolites, including 4-hydroxy tamoxifen (TOH)^13^. TOH is nearly 30 to 100 times more potent than TAM in inhibiting estrogen-dependent cell proliferation^13^. P-gp plays an important role in the active transport of TAM and its metabolite TOH^14^. Being a substrate for CYP3A4 metabolizing enzymes and P-gp/BCRP transporters, TAM is a candidate of many DDIs. The various CYP-450 enzymes (CYP2D6, CYP2C9, CYP3A4) that are involved in TAM metabolism can be altered by many factors either due to variation in pharmacogenesis or due to induction/inhibition by various pharmaceutical drugs or natural products with a possible alteration in TAM bioavailability^15,16^. The effect of different flavonoids on the PK of TAM and TOH was studied in rats^17–21^. Most of the studied flavonoids resulted in a significant increase in the bioavailability of TAM with a significant alteration in metabolite (TOH)/parent (TAM) ratio suggesting flavonoid-related effect on the metabolism of TAM to TOH^17–21^. Accordingly, therapeutic drug monitoring (TDM) has been recommended with TAM therapy for dose individualization to ensure the required efficacy^22^.

In cancer chemotherapy, the most common obstacles hindering efficacy are the toxic effects of chemotherapeutic agents as well as the acquired resistance of cancer cells to the anticancer medications. It has been suggested that the expression of the efflux proteins, P-gp and BCRP, largely contribute to the multidrug resistance and hence poor response to many chemotherapeutic agents^23–26^. Owing to the fact that the overexpression of efflux transporters (P-gp, BCRP) in breast cancer cells to TAM therapy constitutes a major problem in oncology practice^27^, the inhibitors of P-gp/BCR transporters could increase drug exposure, yet with increased side effects^23^. Moreover, TAM and TOH have the potential to inhibit P-gp mediated drug transport as well as CYP3A-mediated metabolism^28^. Accordingly, they could affect the PK of co-administered drugs. Our research team has previously studied the PK interaction between TAM and the anticancer drug erlotinib (ERL)^29^ where an increase in TAM C_max_ with no significant increase in AUC, along with a decrease in AUC of ERL were recorded.

The synergistic antiviral effect of both DSV and TAM suggested their possible co-administration in the management of HCV infections. However, being substrates and inhibitors of efflux transporters (TAM inhibits P-gp, DSV inhibits P-gp and BCRP), there is a possibility of DDIs. Accordingly, an alteration in drug exposure (TAM/TOH/DSV) could constitute a major concern in actual practice.

This work aims at studying-for the first time- the possible PK interaction between TAM and DSV following their co-administration in Wistar rats. Drug plasma concentrations of TAM, the active metabolite TOH, and DSV were measured using newly developed UPLC-MS/MS. This technique was previously reported for the bioanalysis of either TAM/TOH^29–33^ or DSV^34,35^. The proposed method has many advantages over the previously reported methods; it provided lower LLOQ for the determination of TAM/TOH^21,30,31,33^. This enables the determination of very low drug concentrations which is required during the terminal phase elimination. This method also provided shorter analysis time (1.5 min) relative to previously published methods for either TAM^21,31–33^ or DSV^34,35^ determinations. This is extremely important for high throughput analytical methods. This method also has the advantage of utilizing small sample volume, 50 µL plasma, which was significantly less compared with previously reported methods^21,31,34^. Due to the small total blood volume of rats, the small sample volume is considered essential to maintain treated rats in a good health during the whole sampling time (up to 48 h). It is also noteworthy to mention that the proposed method-to the best of our knowledge-is considered the first to describe the simultaneous analysis of TAM/TOH and DSV. The proposed method was fully validated with reference to the FDA guidance for bioanalytical method validation^36^.

## Results and Discussion

### Method development and optimization

#### Chromatographic conditions

Chromatographic conditions were optimized in an attempt to achieve sharp, symmetric, well-quantified peaks, with maximum intensity, and within reasonable runtime. In this respect, mobile phase composition and elution mode was optimized. Different mobile phase systems composed of acetonitrile/water and methanol/water, with or without formic acid, were evaluated. Compared with methanol, acetonitrile produced better peak shape and response, along with lower background noise. Regarding the content of acetonitrile in the mobile phase, it was practically noticed that optimum acetonitrile percent was 80% since it provided the best compromise between peak shape, response, and elution time, for the three compounds. Acetonitrile content of less than 50% resulted in distortion of DSV peaks, along with increased retention for all analytes. Improvement in peak shape and response was achieved with increasing acetonitrile content from 40–90%, with 80% being the most ideal regarding signal intensity and retention time. Formic acid was essential to increase the ionization in the positive ESI mode, hence increasing the signal response. Mobile phases of 80% acetonitrile in water were tried with different proportions of formic acid (0.05–0.15%) and 0.1% formic acid content in the mobile phase was found optimum regarding peak shape and response for the three compounds; DSV, TAM, TOH. Thus, final analysis was performed using isocratic elution with mobile phases consisting of binary mixtures of acetonitrile: water (80: 20, v/v), each with 0.1% formic acid. Under these conditions, the analytes were eluted in the following order, DSV at 0.41 ± 0.02 min, TOH at 0.58 ± 0.03 min, TAM at 0.89 ± 0.03 min, with OND (IS) at 0.51 ± 0.03 min and the total runtime was 1.5 min.

#### Mass spectrometric conditions

For simultaneous determination of DSV, TAM, and its metabolite TOH, different tandem mass spectrometric conditions were optimized. Initially, the ionization mode was optimized. Positive ESI ionization mode yielded higher response for the three analytes, compared with the negative mode, thus it was selected for actual analysis. For quantitation purpose, MRM mode was used to detect the three analytes by monitoring protonated precursor to product ions at *m/z* 494.14 > 359.00 (DSV), *m/z* 372.06 > 72.00 (TAM), *m/z* 388.10 > 71.97 (TOH), and at *m/z* 294.05 > 169.96 (OND, IS). Different detection parameters were optimized to attain the highest response of either protonated precursor ions (e.g. capillary voltage, cone voltage, source temperature, desolvation temperature, and desolvation gas flow rate) or product ions (e.g. collision energy). The source and desolvation temperatures of 150° and 200 °C, respectively, yielded the highest response of protonated precursor ions of the three compounds. Also, operating gases with flow rates of 800 L/h, 150 L/h, and 0.15 mL/min for desolvating, cone, and collision gases, respectively, were optimum for analysis. Cone voltage, capillary voltage, and collision energy were separately optimized for each individual compound and their optimum values were listed in Table 1. Supplementary Fig. S1 shows full scan product ion spectra for the three compounds.

**Table 1:** LC–MS/MS optimized parameters and regression and statistical parameters for the determination of DSV, TAM, and TOH in rat plasma by the proposed method*.

Parameter	DSV	TAM	TOH
Precursor ion [M+H]^+^	494.14	372.06	388.10
Daughter ion	359.00	72.00	71.97
Cone voltage (V)	70	40	70
Capillary voltage (KV)	5.0	4.0	4.0
Collision energy (eV)	35	25	25
Linearity range (ng/mL)	20–1000	0.1–500	0.5–500
LLOQ^a^(ng/mL)	20	0.10	0.50
LLOD^b^ (ng/mL)	10	0.04	0.30
Intercept (a)	−0.0386	0.0147	−0.0106
Slope (b)	0.0234	0.9565	0.2363
Correlation Coefficient (r)	0.9989	0.9989	0.9979
S_a_^c^	0.00722	0.00539	0.00748
S_b_^d^	0.00016	0.00705	0.00194
S_y/x_^e^	0.01033	0.00958	0.01273

*The IS (OND) was measured at *m/z* 294.05 > 169.96, cone voltage of 35 V, capillary voltage of 4.0 KV, and collision energy of 25 eV.

^a^LLOQ: lower limit of quantification. ^b^LLOD: lower limit of detection.

^c^S_a_: standard deviation of intercept. ^d^S_b_: standard deviation of slope.

^e^S_y/x_: standard deviation of residuals.

#### Sample preparation and selection of IS

Different methods are generally used for sample preparation, namely protein precipitation (PPT), liquid-liquid extraction (LLE), and solid-phase extraction (SPE). Moreover, for better sample clean-up, a combinatorial method of PPT and SPE has been proved to be efficient in removing interfering plasma components, decreasing background noise, and providing consistent good recoveries of the analytes from plasma^29,37–40^. Thus, this method (PPT/SPE) was applied in this work. Based on our previous work for the simultaneous analysis of erlotinib and TAM in rat plasma^29^, Strata^TM^ −X-C 33 µm strong cation cartridges (200 mg, 3 mL) (Phenomenex, Torrance, USA) was found optimum since it provided better and consistent extraction recoveries of the analytes, compared with other SPE cartridges which had been tried (octadecyl C 18, octyl C 8, ethyl C 2, and cyanopropyl CN (200 mg, 3 mL) (Spe-ed SPE car-tridges, Applied Separations, Allentown, PA). Being weakly basic compounds (The pKa values of DSV are 8.2 (pK_1_) and 9.2 (pK_2_), pka of TAM is 8.76, pka values of TOH are 8.66, 9.45)^41^, cation mixed mode cartridges, applying both reversed phase and ionic interactions for analyte retention, produced efficient recovery of the three analytes. Moreover, we previously reported that, following experimental trials, the optimum operating procedure for TAM extraction was as follows^29^; 50 µL plasma samples were spiked with the analytes and the IS, treated with 150 µL 2% formic acid, and then completed to 1-mL using acetonitrile for PPT. Following centrifugation, further purification was performed using Strata^TM^ −X-C 33 µm SPE cartridges which had been preconditioned with 3 mL water followed by 3 mL 2% formic acid. Next to sample loading, the cartridges were washed with 1.0 mL 2% formic acid. Retained analytes were then eluted using 1.0 mL 5% ammonia solution in acetonitrile. The eluted solutions were evaporated to dryness and reconstituted into 0.5 mL acetonitrile before actual analysis. Using the optimized procedure, good recoveries (error values of less than 20% for LLOQ and less than 15% for higher concentrations) were recorded for the three analytes; DSV, TAM, and TOH.

OND was selected as the IS in the determination of DSV, TAM, and TOH. OND showed a similar chromatographic behavior to the analyzed compounds with comparable response and retention time. Moreover, it could be detected in the positive ionization mode used for detecting the analytes.

### Method validation

The following parameters were evaluated: specificity, linearity, lower limits of detection and of quantification, precision, accuracy, stability, dilution integrity, and carryover effect.

#### Selectivity

**Figure 2:**
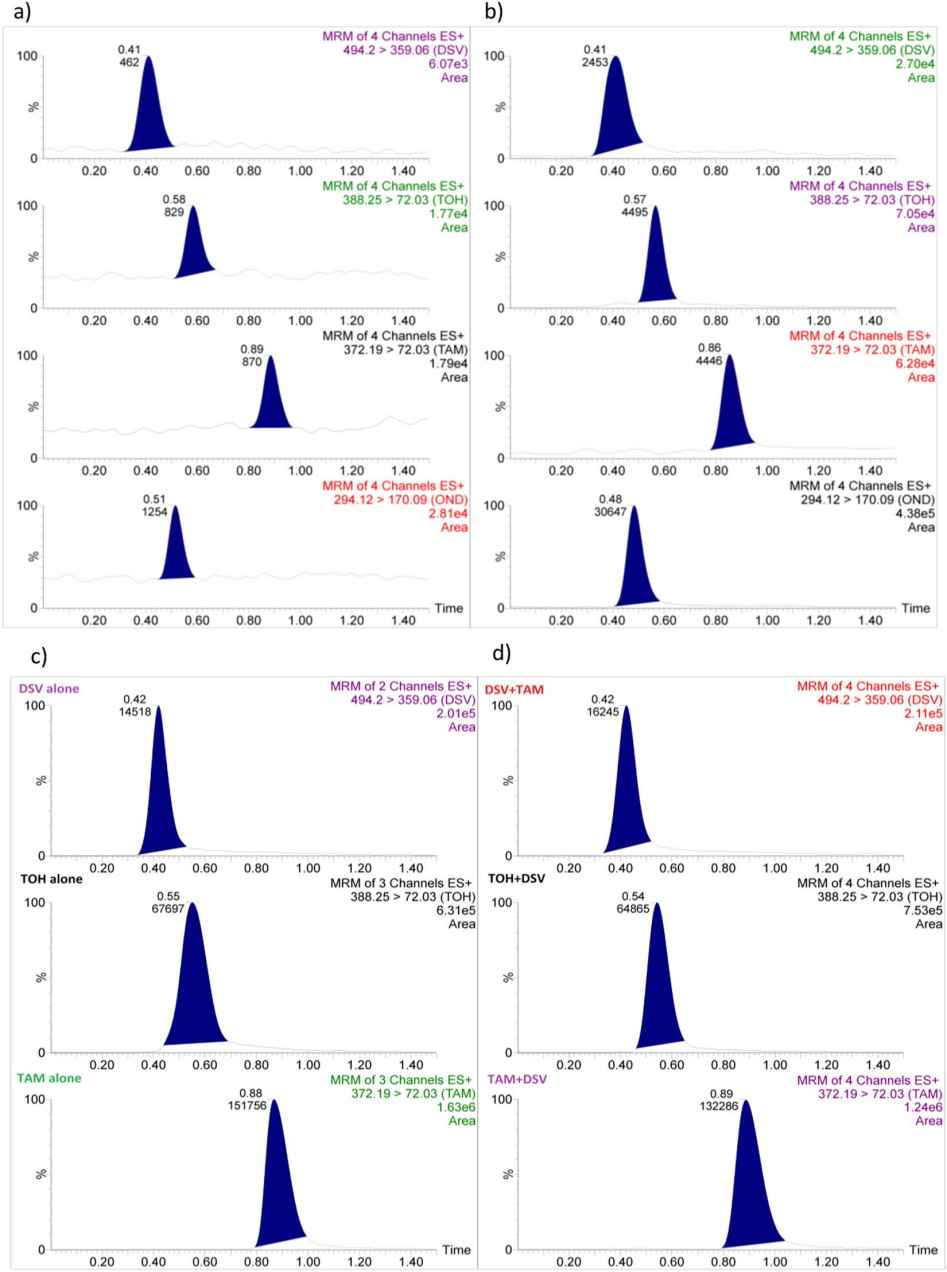
Multiple reaction monitoring (MRM) of a blank plasma, (**a**), and a plasma sample spiked with a standard mixture of DSV, TAM, and TOH at their LLOQ level with OND (IS), (**b**), and of rat plasma samples 1 h following single administration of DSV (50 mg/kg)/TAM (10 mg/kg), compared with their co-administration, (**d**).

Figure 2 shows the absence of interference at the retention times of DSV, TAM, and TOH. It was clear that the analytical signals at LLOQ of the analytes were at least five times as much as those recorded from blank samples, at the same retention times. Moreover, signals of IS were at least twenty times of that of the blank.

#### Linearity

Linearity was assessed using eight matrix-based calibration standards within the concentration range 20–1000 ng/mL (DSV), 0.1–500 ng/mL (TAM), and 0.5–500 ng/mL (TOH). The method of least squares was used to relate the peak area ratio of each analyte to the IS and the corresponding spiked concentration. Table 1 shows linearity and statistical parameters for the simultaneous determination of the three analytes using the proposed method. Theses parameters include; correlation coefficients (r), intercepts (a), slopes (b), standard deviations of the intercept (S_a_), standard deviation of the slope (S_b_), and standard deviation of residuals (S_y/x_). The high values of the correlation coefficients, r ≥ 0.9979, for the three analytes, along with the small intercepts, indicated high degree of linearity of the proposed method.

#### Lower limit of detection (LLOD) and lower limit of quantification (LLOQ)

LLOD and LLOQ for the three analytes were selected based on the criteria that concentrations at LLOD should provide analytical signals of at least three times that of the blank signal while concentrations at LLOQ should yield signals of at least five times as much as blank signals and they should also be quantified with acceptable accuracy (error values ≤ 20%) and precision (RSD values ≤ 20%). Accordingly, LLOD were found to be 10, 0.04, 0.30 ng/mL, while LLOQ were 20, 0.1, and 0.5 ng/mL, respectively (Table 1). MRM chromatograms of plasma samples spiked with the three analytes at their LLOQ, compared with blank samples, were shown in Fig. 2. The results revealed that the proposed method provided low LLOQ values. This allows the extension of the method for trace analysis of the three analytes, an important aspect in TDM, during actual administration of DSV/TAM to human subjects.

#### Extraction recovery

Extraction recoveries of the three analytes from plasma samples were calculated at the four quality control (QC) concentration levels (Table 2). The obtained recoveries ranged from 90.11–97.05 (DSV), 89.71–97.40 (TAM), and 91.95–101.68 (TOH). In addition, OND (IS) provided an acceptable recovery of 93.82%, evaluated at the concentration used in actual analysis. Table 2 ensures the validity of sample treatment procedure for proper extraction of the analytes from plasma samples.

**Table 2:** Evaluation of the extraction recovery, matrix effect, and precision in the UPLC-MS/MS analysis of standard mixtures of DSV, TAM, and TOH with OND (IS).

	Concentration spiked (ng/ml)	Extraction recovery		Matrix effect		Precision			
						Intra-day (n = 6)		Inter-day (n = 18)	
		Mean recovery (%) ± RSD^a^	E_r_(%)^b^	Mean recovery (%) ±RSD^a^	E_r_(%)^b^	Mean recovery (%) ±RSD^a^	E_r_(%)^b^	Mean recovery (%) ±RSD^a^	E_r_(%)^b^
DSV	20	96.37 ± 1.66	−3.63	97.78 ± 2.32	−2.22	98.60 ± 1.19	−1.40	97.43 ± 2.06	−2.57
	60	97.05 ± 1.59	−2.95	97.28 ± 2.45	−2.72	97.22 ± 1.92	−2.78	98.65 ± 1.89	−1.35
	500	96.30 ± 1.47	−3.70	93.65 ± 4.56	−6.35	98.21 ± 1.95	−1.79	98.12 ± 0.66	−1.88
	800	90.11 ± 6.38	−9.89	99.32 ± 1.85	−0.68	97.67 ± 2.17	−2.33	97.77 ± 1.03	−2.23
TAM	0.1	95.05 ± 2.51	−4.95	96.21 ± 1.32	−3.79	95.58 ± 3.28	−4.42	93.70 ± 2.24	−6.30
	0.3	97.40 ± 2.47	−2.60	96.66 ± 1.83	−3.34	98.57 ± 0.79	1.43	97.89 ± 1.52	−2.11
	100	96.48 ± 1.12	−3.52	92.26 ± 3.01	−7.74	99.07 ± 3.98	−0.93	99.13 ± 0.76	−0.87
	400	89.01 ± 7.88	−10.99	98.89 ± 7.54	−1.11	98.47 ± 2.44	−1.53	98.77 ± 1.75	−1.23
TOH	0.5	101.68 ± 2.33	1.68	97.96 ± 0.99	−2.04	99.95 ± 3.04	−0.05	98.08 ± 1.43	−1.92
	1.5	95.82 ± 5.65	−4.18	97.73 ± 1.28	−2.27	99.41 ± 2.04	−0.59	99.65 ± 1.74	−0.35
	150	99.78 ± 0.99	−0.22	99.02 ± 2.44	−0.98	96.36 ± 3.05	−3.64	98.80 ± 1.57	−1.20
	400	91.95 ± 3.21	−8.05	101.45 ± 2.78	1.45	98.01 ± 2.02	−1.99	97.25 ± 2.61	−2.75

^a^Mean recovery (%) ± RSD of six determinations.

^b^Percentage relative error.

#### Matrix effect

Matrix effect was evaluated at the same concentration levels of the three analytes as those used for evaluating the extraction recovery. The obtained values were shown in Table 2 where error values did not exceed 6.35 (DSV), 7.74 (TAM), and 2.27 (TOH). Additionally, the effect of matrix on the analysis of OND (IS) at its applied concentration did not exceed 6.89. The obtained results revealed that matrix effect of the proposed method was negligible, thus allowing trace analysis of the analytes in actual samples.

#### Precision and accuracy

Precision (in terms of RSD) and accuracy (in terms of E_r_%) were evaluated at two levels; intra-day and inter-day, using the four QC concentration levels of the three analytes. Intra-assay results revealed that error values did not exceed 4.42, while deviation values were not more than 3.98. For inter-assay analysis, the calculated errors and deviation values did not exceed 6.30 and 2.61 for DSV, TAM, and TOH, respectively. All results were presented in Table 2. The method yielded acceptable degree of accuracy and precision for the determination of the three analytes since all obtained error and deviation values did not exceed the permitted limits; 20% for LLOQ and 15% for higher concentrations.

#### Dilution integrity

The integrity of the three analytes was assessed up to five-fold dilutions. Table 3 shows that diluted samples yielded acceptable recovery and deviation values, all values did not exceed the acceptance limits of ±15%.

**Table 3:** Evaluation of the dilution integrity of DSV, TAM, and TOH in rat plasma.

	Concentration spiked (ng/mL)	Dilution fold	Mean recovery (%) ± RSD^a^	E_r_(%)^b^
DSV	2000	1:2	98.09 ± 2.69	−1.91
		1:5	99.74 ± 3.96	−0.26
TAM	1000	1:2	98.07 ± 1.58	−1.93
		1:5	96.11 ± 2.53	−3.89
TOH	1000	1:2	97.15 ± 1.64	−2.85
		1:5	95.44 ± 0.78	−4.56

^a^Mean recovery (%) ± RSD of six determinations.

^b^Percentage relative error.

#### Stability studies

The stability of DSV, TAM, and TOH were evaluated at different storage and treatment conditions as mentioned under the experimental section. Table 4 shows that all obtained recovery values (±RSD) were within the acceptable limits (±15%). This ensures that no detectable degradation was noticed and that all analytes were stable under the studied conditions. The analytes’ solutions were also stable when kept refrigerated at 4 °C for 3 months or at room temperature for up to 6 h.

**Table 4:** Evaluation of the stability of DSV, TAM, and TOH in rat plasma.

Stability	Concentration level*	Mean recovery (%) ± RSD^a^		
		DSV	TAM	TOH
Auto-sampler stability (10 °C, 56 h)	Low	95.46 ± 1.77	98.25 ± 2.56	99.67±1.35
	High	98.49 ± 0.72	93.46 ± 2.43	95.15 ± 5.66
Short-term stability (25 °C, 6 h)	Low	96.35 ± 3.32	98.22 ± 1.21	96.88 ± 2.77
	High	95.05 ± 2.68	96.77 ± 2.85	98.90 ± 0.52
Long-term stability (−30 °C, 30 days)	Low	96.82 ± 3.62	99.69 ± 1.04	99.03 ± 3.19
	High	98.82 ± 2.60	97.30 ± 4.59	95.82 ± 1.22
Freeze-thaw stability (−30 °C, 3 cycles)	Low	98.51 ± 0.49	96.11 ± 3.21	97.49 ± 2.09
	High	98.54 ± 1.59	93.93 ± 6.05	97.59 ± 0.63
Refrigerator (4 °C, 3 months)	Low	96.23 ± 3.54	98.91 ± 2.09	97.99 ± 5.06
	High	97.57 ± 2.10	96.61 ± 2.84	97.03 ± 0.95

*Two concentration levels were evaluated for each analyte; low (200, 1, 5 ng/mL for DSV, TAM, and TOH, respectively) and high (800, 400, 400 ng/mL for DSV, TAM, and TOH, respectively).

^**a**^Mean recovery (%) ± RSD of six determinations.

#### Carry over

The analyte’ peaks observed in the blank samples which had been injected directly following the highly concentrated plasma samples were of peak areas less than 20%, compared with LLOQ, of each analyte. Moreover, the mean carryover of the IS was lower than 5% of the IS. These results showed that the carryover effect is negligible for the analysis of the three analytes by the proposed method.

### Pharmacokinetic interaction studies

**Figure 3:**
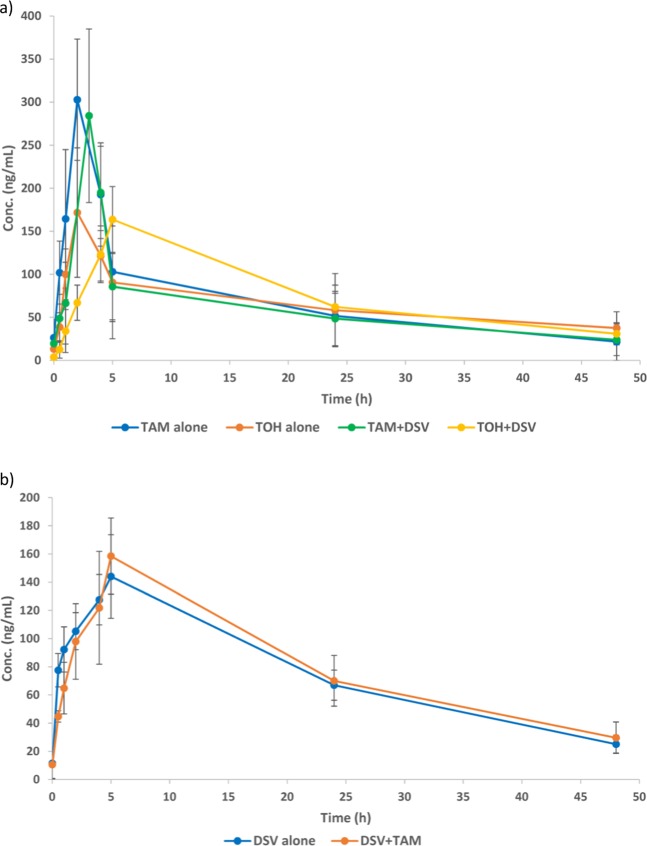
Plasma concentration– time profile of TAM, TOH, (**a**), and DSV, (**b**), in rats after an oral administration of a combination of DSV (50 mg/kg) and TAM (10 mg/kg), compared with their single administration. Suitable dilutions of prepared plasma samples were made before analysis.

The combination of TAM with DSV could be of therapeutic importance because of their possible synergistic antiviral effect. However, review of the literature revealed that-to our knowledge- no study was concerned with their PK interaction. In this work, the applicability of the proposed UPLC-MS/MS method was extended to studying-for the first time- the possibility of the PK interaction between DSV and TAM in Wistar rats. TOH is the active metabolite of TAM and it has reported estrogenic properties being at least 30–100 times more potent than TAM. Thus, the developed method was concerned with the determination of TOH, besides TAM, the parent drug. Figure 3 shows the mean plasma concentration-time profiles of DSV, TAM, TOH following the co-administration of DSV and TAM, compared with their single oral administration. Different PK parameters were derived from NCA of plasma concentration/time data as listed in Table 5. The results revealed that no significant changes were recorded in any of the PK parameters for both DSV and TAM/TOH following their co-administration (Group III), as compared with their single administration (Group I for DSV and Group II for TAM). The only exception was for t_max_ of TOH.

**Table 5:** Main pharmacokinetic parameters (mean ± SD) following oral single administration of either DSV/TAM to rats, compared with their co-administration (*n* = 5).

	Single administration			Co-administration		
	DSV	TAM	TOH	DSV	TAM	TOH
C_max_ (ng/mL)	144.04 ± 26.50	306.97 ± 108.56	171.62 ± 54.00	158.98 ± 49.00	287.52 ± 81.05	163.67 ± 54.50
t_max_ (h)	4.95 ± 1.07	2.03 ± 0.95	2.03 ± 0.95	4.98 ± 0.61	3.03 ± 0.66	5.03 ± 0.66*
t_½_ (h)	17.95 ± 5.93	15.75 ± 5.18	28.85 ± 1.98	19.72 ± 5.41	17.67 ± 3.42	19.72 ± 1.41
AUC_0-∞_ (ng.h/mL)	4268.14 ± 754.40	3878.94 ± 406.62	4727.49 ± 774.18	4662.32 ± 686.97	3507.87 ± 463.83	4547.03 ± 913.49
CL/F (mL/h/kg)	1.70 ± 6.81	2.62 ± 1.53	2.12 ± 1.03	10.89 ± 7.49	2.82 ± 0.88	2.19 ± 0.46
R.B.(%)a	—	—	—	109 ± 11.50	90 ± 8.55	96 ± 9.22
M.R.(%)b	—	—	121.86 ± 11	—	—	129.65 ± 10.88

*Indicates significant difference as compared with the corresponding single administration; group I for DSV, group II for TAM and TOH. (p = 0.05)

^a^Relative bioavailability calculated by the ratio of AUC_0-∞_of each drug following co-administration of DSV and TAM, as compared with the single administration.

^b^Metabolite ratio calculated by the ratio of AUC_0-∞_ of TOH to that of TAM following co-administration of DSV and TAM, as compared with the single TAM administration.

The study design was based on that although DSV has no reported modulatory effect on CYP enzymes, yet it is a potent inhibitor of the efflux proteins P-gp/BCRP. Thus, it was expected to inhibit TAM/TOH efflux with a subsequent increase in the bioavailability since P-gp/BCRP-mediated efflux constitutes a major barrier to TAM/TOH systematic exposure with development of multidrug resistance. However, *in-vivo* experiments revealed that the relative bioavailability percentage (R.B. %) of neither TAM nor TOH was affected by DSV administration. Moreover, DSV had no significant effect on the degree of TAM metabolism since no significant change (p > 0.05) in metabolite/parent ratio (M.R. %) was observed.

Similarly, TAM/TOH has an inhibitory effect on CYP3A4 enzymes and P-gp transporters. Giving that DSV is metabolized mainly by CYP2C8 and to a lesser extent by CYP3A4 and that it is transported mainly by P-gp transporters, TAM was expected to increase DSV exposure through P-gp inhibition and CYP3A4 inhibition. Yet, the animal experiments revealed the absence of any significant effect of TAM on any of the measured PK parameters of DSV.

The obtained results suggest the safety of DSV/TAM antiviral combination and the possibility of their co-administration without the need for dosage adjustment. Since rats were selected to carry out the study and due to possible PK changes when shifting to the human level, further clinical studies on human subjects should be conducted to emphasize the obtained results.

An analogous result was obtained with the PK interaction study between TAM/TOH when co-administered with biochanin A, a potent inhibitor of CYP3A and P-gp/BCRP transporters^21^. The study revealed that biochanin A did not significantly change the PK behavior of TAM or its metabolite TOH. Many unexpected results were also noticed with DSV. Absence of DDIs between co-administered medications was reported in cases where DDIs had been postulated. This again stresses on the mystery of DDIs and despite being postulated theoretically in many cases, still *in-vivo* studies should be conducted to investigate the actual situation.

## Conclusion

In this study, UPLC-MS/MS method has been developed and validated for the simultaneous determination of TAM, its metabolite TOH, and DSV in rat plasma. Sample preparation was carried out using PPT followed by SPE with mixed-mode cationic cartridges. Full validation of the proposed method was performed with reference to the FDA guidelines. The method was successfully applied to study the PK interaction between TAM and DSV in combination, compared with their monotherapy. No significant change in the PK of any of the measured compounds (TAM/TOH, DSV) was noticed following the co-administration of TAM/DSV combination suggesting the safety of this combination with no prior dose adjustment.

## Experimental

### Materials and reagents

DSV reference standard was obtained from Haoyuan Chemexpress Co., Ltd., (Shanghai, P.R.China). Reference standards of TAM and TOH were purchased from Sigma Aldrich Co. (St. Louis, MO, USA). Ondansetron (OND), used as the internal standard (IS), was also purchased from Sigma Aldrich Co. (St. Louis, MO, USA). All reference standards were supplied with purity ≥98%.

HPLC grade methanol and acetonitrile (Panreac, E.U.) were used in the study. Formic acid was obtained from Sigma Aldrich Co. (Chemie GmbH, Steinheim, Germany). Ammonium hydroxide solution (30–35%) was supplied from Winlab Laboratory Chemicals (Leicestershire, UK). Ultrapure Milli-Q Advantage water (Millipore, Molsheim, France) was used in the study.

### Instrumentation

The UPLC-MS/MS instrument (Waters Model Xevo TQ-S, Singapore, Singapore) was equipped with sample manager and binary solvent manager (Acquity ^™^ Ultra-performance LC). Detection was performed with a triple-quadrupole mass spectrometric detector (STEP WAVE ^™^, Ultra-performance LC) supplied with different ionization sources (Zspray ^™^ ESI-APCI-ESCI, Ultra-performance LC). Multiple reaction monitoring (MRM) detection mode was applied in the study. System operation and data processing were performed using Masslynx^TM^ software, Version 4.1 (Micromass, Manchester, UK).

For Sample preparation, strong cation solid-phase extraction (SPE) tubes (Strata^TM^ -X-C 33 µm, 200 mg/3 mL), obtained from phenomenex (Torrance, USA), were used. Solvent evaporation was performed using nitrogen evaporator (N-EVAP 112) with a heating system (OA-SYS) supplied from Organomation Assocciates, Inc. (Berlin, Massachusetts, USA). All samples were filtered using disposable syringe filters (CHROMAFIL^®^ Xtra PA-20/25 polyamide filters, 0.2 µm pore size) obtained from MACHEREYNAGEL, GmbH & Co. (KG, Duren, Germany).

### Operating LC-MS/MS conditions

Chromatographic analysis was performed using Acquity UPLC BEH^™^ C 18 analytical column (100 ×1.0 mm, i.d., 1.7 µm particle size) (Waters, Dublin, Ireland) which was maintained at 45 °C. Isocratic elution was performed with a mobile phase consisting of a mixture of water and acetonitrile, each with 0.1% formic acid, in the ratio of 20: 80, v/v. The flow rate of 0.2 mL/min was used all over the run (1.5 min). Samples were introduced into the system using the auto-sampler, set at 10 °C, operated in the partial loop mode and with injection volumes of 5 µL.

Tandem mass spectrometric detection was performed using positive electrospray ionization (ESI) mode. The source and desolvation temperatures were set at 150° and 200 °C, respectively. Flow rates of operating gases were adjusted at 800 L/h for desolvating gas, 150 L/h for cone gas, and 0.15 mL/min for collision gas. Cone voltage, capillary voltage, and collision energy were operated at the optimized values as mentioned in Table 1. The dwell time was set at 0.025 s. The resolution of the analyzer was operated at 2.8 for low mass (LM) and 14.86 for high mass (HM). MRM of the transitions from protonated precursor ions [M+H]^+^ to selected product ions were used for the quantitation of DSV at *m/z* 494.14 > 359.00, TAM at *m/z* 372.06 > 72.00, TOH at *m/z* 388.10 > 71.97, and with OND (IS) detected at *m/z* 294.05 > 169.96 (Table 1).

### Preparation of stock and standard solutions

Stock solutions of 1 mg/mL of TAM, TOH, and OND (IS) were prepared in methanol. For DSV, stock solutions of 1 mg/mL were prepared in water/acetonitrile (20: 80, v/v). Acetonitrile was used for further dilutions to prepare standard solutions of DSV, TAM, and TOH of suitable concentrations. A standard solution of 50 ng/mL OND was used for spiking plasma samples.

### Preparation of calibration standards and quality control samples

Matrix-based calibration standards were prepared by spiking 50 µL volumes of drug-free rat plasma samples with suitable volumes of DSV, TAM, and TOH standard solutions to prepare eight calibration standards within the concentration ranges of 20–1000 ng/mL (DSV), 0.1–500 ng/mL (TAM), and 0.5–500 ng/mL (TOH). A fixed volume of 50 µL of OND (IS) (50 ng/mL) was added to each sample. To each spiked sample, 150 µL of 2% formic acid was added and then completed to a final volume of 1-mL with acetonitrile. Blank samples were prepared by mixing 50 µL volumes of drug-free plasma with 150 µL of 2% formic acid before the final dilution to 1-mL volume with acetonitrile. By analogy, QC samples were prepared at four different concentration levels within the linearity range of each compound. These concentrations were as follows: very low concentrations (LLOQ, 20 ng/mL, 0.1 ng/mL, and 0.5 ng/mL for DSV, TAM, and TOH, respectively), low concentrations (60 ng/mL, 0.3 ng/mL, and 1.5 ng/mL for DSV, TAM, and TOH, respectively), medium (500 ng/mL, 100 ng/mL, and 150 ng/mL for DSV, TAM, and TOH, respectively), and high (800 ng/mL, 400 ng/mL, and 400 ng/mL for DSV, TAM, and TOH, respectively).

### Sample preparation

All plasma samples (blank and spiked) were vortex-mixed at 6000 rpm for 5 min then centrifuged for 15 min. The centrifugate was then poured onto Strata^TM^ −X-C 33 µm SPE tubes which had been previously preconditioned with 3.0 mL methanol followed by 3.0 mL 2% formic acid. The cartridges were washed with 1 mL of 2% formic acid. The retained analytes were finally eluted with 1.0 mL 5% ammonium hydroxide solution in acetonitrile. The collected eluents were evaporated to dryness under nitrogen and the obtained residue was reconstituted in 0.5 mL acetonitrile and then injected into the UPLC-MS/MS system for actual analysis.

### Method validation

#### Specificity

Blank plasma samples from six different batches were used to assess specificity. Comparison was performed between the chromatograms obtained from the analysis of blank plasma samples and those of plasma samples spiked with LLOQ of DSV (10 ng/mL), TAM (0.1 ng/mL), and TOH (0.5 ng/mL). For all samples, the response signals at the retention times of the three drugs, along with OND (IS), were recorded.

#### Linearity

Calibration graphs were constructed using plasma samples spiked with the three drugs at eight different concentrations in the range 20–1000, 0.1–500, and 0.5–500 ng/mL for DSV, TAM, and TOH, respectively. Volumes of 50 µL OND (50 ng/mL), as IS, were separately added to each sample. Method of least squares was used to relate the peak area ratios of each of the three drugs to OND (IS) and consequently the regression equations were derived.

#### Lower limit of detection (LLOD) and lower limit of quantification (LLOQ)

Both LLOD and LLOQ were practically evaluated. Concentrations giving analytical responses of at least three times or five times the blank signal, measured at the same retention times of the compounds, were taken as LLOD and LLOQ, respectively. The concentrations taken as LLOQ should provide errors and relative deviation values of not more than 20% assessing acceptable accuracy and precision.

#### Extraction recovery

Extraction recovery was assessed by analyzing QC samples prepared at four QC concentration levels (20, 60, 500, 800 ng/mL for DSV, 0.1, 0.3, 100, 400 ng/mL for TAM, 0.5, 1.5, 150, 400 ng/mL for TOH) in six replicates. For evaluating the extraction recoveries of the three analytes (DSV, TAM, TOH) from plasma samples, peak responses of each analyte from QC samples spiked pre-extraction were compared with those spiked post-extraction. In addition, the recovery of OND (IS) was evaluated at the concentration level employed in the analysis.

#### Matrix effect

For matrix effect, calculations were based on comparing the peak responses from the four QC samples, used for evaluating the “extraction recovery”, spiked post-extraction with those of standard acetonitrile drug solutions prepared of the same nominal concentrations.

#### Precision and accuracy

Precision and accuracy were assessed by analyzing QC samples prepared at the four concentration levels, as used for evaluating the extraction recovery. Sample analysis was performed six times on the same day or on three successive days for intra-day and inter-day precision and accuracy, respectively. Found concentrations were calculated by relating peak area ratio of each drug to the IS with that obtained using freshly prepared calibration standards. Accordingly, percentage relative standard deviation (%RSD) was used to assess precision while the percentage relative error (E_r_%) was used to assess accuracy.

#### Dilution integrity

Dilution integrity was evaluated by analyzing plasma samples spiked with high concentrations beyond the linearity range of the three analytes (2000 ng/mL for DSV and 1000 ng/mL for TAM/TOH) after being diluted with (1: 2) and (1: 5) fold dilution.

#### Stability studies

The stability of the three analytes in plasma samples was assessed by analyzing six replicates of QC samples prepared at two concentration levels, low (60, 0.3, 1.5 ng/mL) and high (800, 400, 400 ng/mL) for DSV, TAM, and TOH, respectively, by calculating the percentage recoveries. Samples were subjected to different storage conditions. Auto-sampler stability was assessed by leaving prepared samples in the auto-injector (10 °C, 56 h) before the injection. Short-term (bench top) stability was evaluated by analyzing samples after being stored at room temperature (25 °C, 6 h). On the other hand, long-term stability was assessed by analyzing samples which had been frozen before analysis (−30 °C, 30 days). Stability of samples was also assessed following three freeze–thaw cycles (frozen at −30 °C, thawed at 25 °C).

#### Carryover effect

The carryover effect was investigated by injecting three blank plasma samples following the injection of plasma samples spiked with high drug concentrations (1000 ng/mL for DSV and 500 ng/mL for TAM and TOH). Peak areas of blank samples were recorded at the retention times of the three analytes and the IS.

### Application to pharmacokinetic interaction studies

#### Compliance with ethical standards

All experiments were carried out in accordance with the ethical guidelines for experimental studies with animals according to the Research Ethics committee, King Saud University. We confirm that the experimental protocol was approved by the Research Ethics committee, King Saud University (Ethics Reference No. KSU-SE-19–13).

#### Animal handling

Male Wistar rats (220–280 g) were obtained from the animal house, Women Student-Medical studies & Sciences Sections, College of Pharmacy, King Saud University (Riyadh, Saudi Arabia). The rats were acclimatized for seven days before starting the experiment. All rats were kept in a well-ventilated room and under standard laboratory conditions; 12 h day/night cycle, average temperature of 25 ± 2 °C, and average relative humidity of 50 ± 2%.

#### Study design

Three group of randomly distributed rats (n = 5) were used to conduct the study. Treated rats were subjected to oral administration of DSV (50 mg/kg), group I, tamoxifen (10 mg/kg), group II, and a combination of DSV (50 mg/kg) and TAM (10 mg/kg), group III. For this purpose, DSV (50 mg/mL) and TAM (10 mg/mL) suspensions were prepared in aqueous solution of carboxy methyl cellulose (0.5% w/v CMC). Based on the group number, rats were separately given volumes of 0.25 mL of DSV/TAM suspension using an oral gavage needle. Following drug administration, orbital blood samples (0.3 mL) were collected into a series of 1.5 mL polythene heparinized tubes prior to dosing (0 time) and at different time intervals following the drug administration; 0.25, 0.5, 1, 2, 4, 5, 12, 24, and 48 h. Immediate centrifugation (4,500 rpm) of collected blood samples was performed at 4 °C for 30 min. The resulting plasma samples were then stored at −20 °C until the day of analysis. Plasma samples (50 µL) were separately mixed with 50 µL OND, IS, (50 ng/mL), 150 µL of 2% formic acid, and finally completed to 1-mL volume with acetonitrile. Sample preparation was then carried out exactly as mentioned under the experimental section. Drug concentrations of DSV, TAM, TOH were computed using matrix-based calibration data. These concentrations were then plotted versus the corresponding withdrawal time.

### PK analysis

Plasma concentration-time data were treated by the non-compartmental analysis (NCA) method using PKSolver Add-In Excel 2010. Different PK parameters were calculated. They included maximum drug plasma concentration (C_max_) and the time required to reach the maximum plasma concentration (t_max_). Both C_max_ and t_max_ were derived from plasma concentration-time curve simply by visual inspection of the data. Using the PKSover, the elimination rate constant (K_el_), the half-life (t_1/2_), the area under the plasma concentration-time curve from 0 time to the last sampling time t (AUC_0-t_), the area under the plasma concentration-time curve from 0 time to ∞ (AUC_0-∞_), and the apparent oral clearance (CL/F) were calculated. K_el_ was calculated from the slope of the plasma concentration-time curve during the elimination phase and from which t_1/2_ was derived, t_1/2_ = 0.693/K_el_. The linear trapezoidal rule was used to calculate AUC_0-t_. The summation of AUC_0-t_ and the extrapolated area from time t to ∞, calculated from the last measured concentration (C_last_), result in total AUC from 0 time to ∞, AUC_0-∞_, AUC_0-∞_ = AUC_0-t_ + (C_last_/K_el_). CL/F was calculated by dividing the dose by AUC_0-∞_. The relative bioavailability (R.B.) was obtained from the ratio of AUC_0-∞_ of the treated group to AUC_0-∞_ of the control group. To investigate the effect of DSV on TAM metabolism, metabolite/parent ratio (AUC_0-∞_), M.R., was calculated by relating AUC_0-∞_ of TOH to that of TAM, presented as percentage^17^.

All PK data were presented as mean ± SD. Statistically significant differences of data from two sets were compared using Student’s t-test where P < 0.05 was used.

## Supplementary information

Supplementary Fig. 1S.
