# Supplementary material for: Development and validation of UPLC-MS/MS method for studying the pharmacokinetic interaction of dasabuvir and tamoxifen, 4-hydroxytamoxifen in Wistar rats
Source: Sci Rep. 2020 Feb 26;10:3521. doi: 10.1038/s41598-020-60613-2 (PMC7044166; doi:10.1038/s41598-020-60613-2)
Supplement: Supplementary file 1 — Supplementary Fig. 1S. [file 41598_2020_60613_MOESM1_ESM.pdf]

Development and validation of UPLC-MS/MS method for studying the pharmacokinetic interaction of dasabuvir and tamoxifen, 4-hydroxytamoxifen in Wistar rats

Aliyah Almomen[1], Hadir M. Maher*[1, 2], Nourah Z. Alzoman[1], Shereen M. Shehata [1], Shorog M. Al-taweel[1], Ashwaq A. Alanazi[1]

[1] College of Pharmacy, Department of Pharmaceutical Chemistry, King Saud University,

Riyadh 11495, P.O. Box 22452, Saudi Arabia.

[2] Faculty of Pharmacy, Department of Pharmaceutical Analytical Chemistry, University of Alexandria, El-Messalah, Alexandria 21521, Egypt

* Correspondence to: H. M. Maher, College of Pharmacy, Department of Pharmaceutical Chemistry, King Saud University, Riyadh 11495, P.O. Box 22452, Saudi Arabia. E-mail: hadirrona@yahoo.com

a)

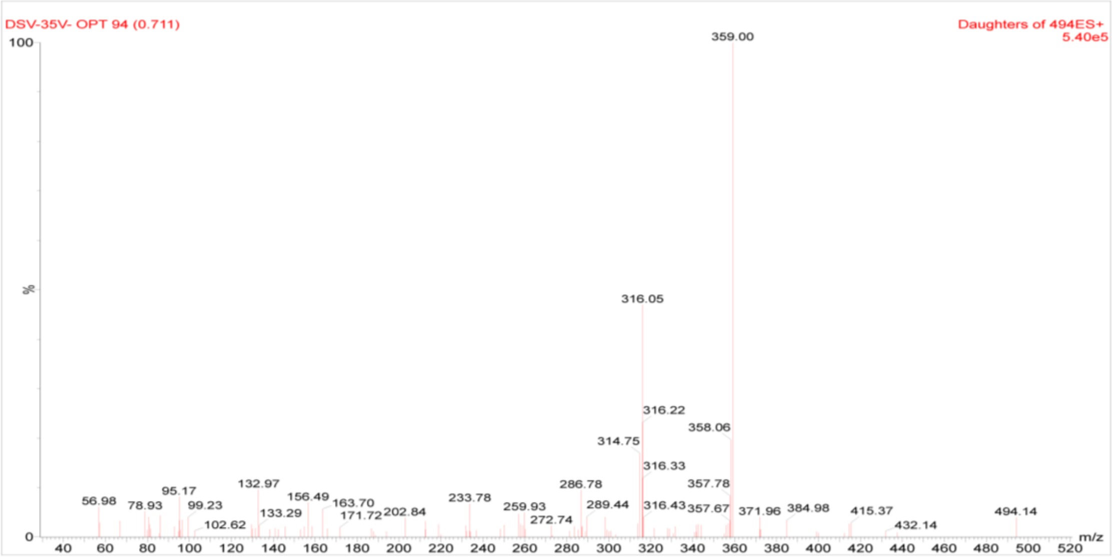
DSV-35V- OPT 94 (0.711)
Daughters of 494ES+
5.40e5
100
%
0
359.00
316.05
316.22
358.06
314.75
316.33
357.78
286.78
132.97
95.17
156.49
56.98
78.93
99.23
163.70
233.78
259.93
289.44
316.43
357.67
371.96
384.98
415.37
494.14
133.29
171.72
202.84
272.74
102.62
432.14
m/z
40 60 80 100 120 140 160 180 200 220 240 260 280 300 320 340 360 380 400 420 440 460 480 500 520

b)

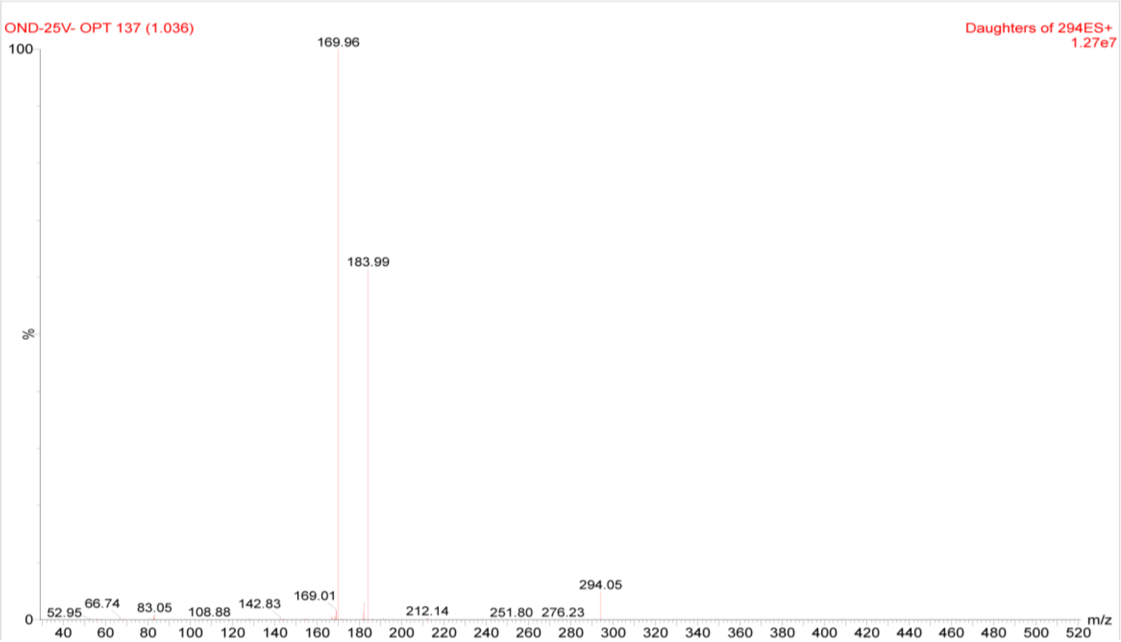
OND-25V- OPT 137 (1.036)
Daughters of 294ES+
1.27e7
100
%
0
169.96
183.99
294.05
169.01
66.74
142.83
52.95
83.05
108.88
212.14
251.80
276.23
m/z
40 60 80 100 120 140 160 180 200 220 240 260 280 300 320 340 360 380 400 420 440 460 480 500 520

c)

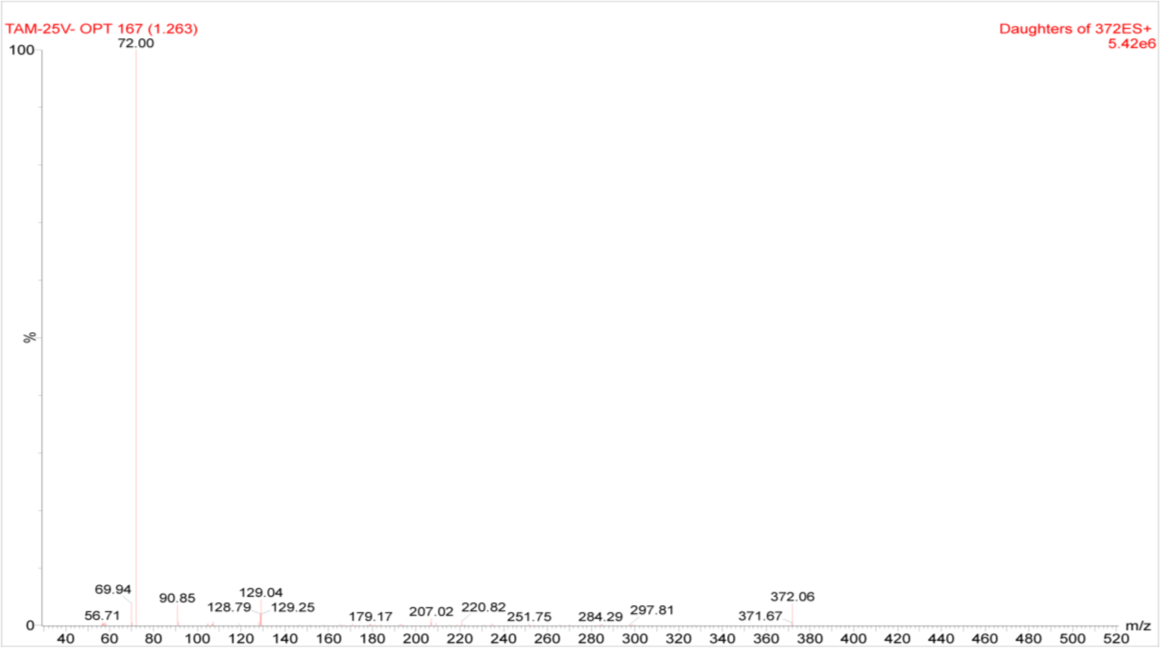

d)

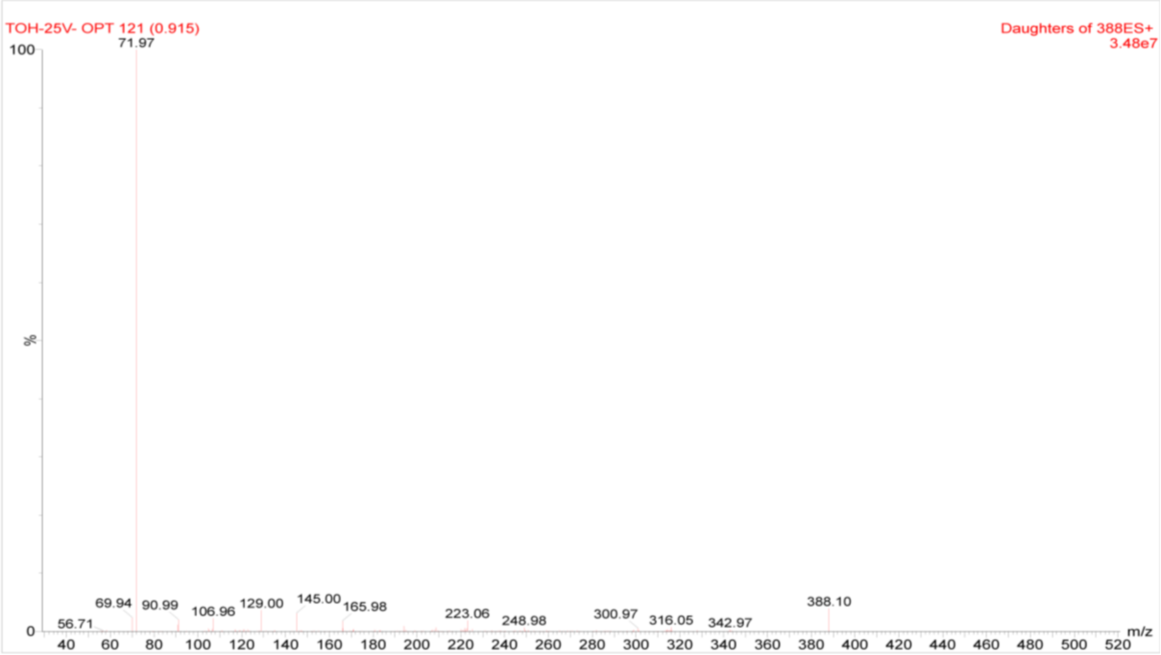

Fig. 1S Product ion spectra of DSV, a), OND, b), TAM, c), and TOH, d).
